# Infantile mesenchymal hamartoma of the liver with elevated alpha fetoprotein

**DOI:** 10.1259/bjrcr.20200196

**Published:** 2021-01-28

**Authors:** Liem Thanh Le, Hai Thanh Phan, Trung Sao Nguyen, Khai Dinh Truong, Dang Thanh Nguyen, Toan Bao Nguyen, Thuy Thi Thu Pham

**Affiliations:** 1 Medic Medical Center, Ho Chi Minh, Vietnam; 2 Medical University, Ho Chi Minh, Vietnam

## Abstract

Mesenchymal hamartoma of the liver (MHL) is a benign tumour that most commonly occurs in children. In most cases of MHL, the α fetoprotein (AFP) level is within the normal limits, only in a few cases, increased AFP has been described which usually causes misdiagnosis of hepatoblastoma. We report a case of a 3-month-old paediatric patient who was incidentally detected with a very high level of AFP, at 6388.4 ng ml^−1^. Ultrasound revealed a right liver tumour, segment VI, measuring at 56 × 53 mm. According to images of ultrasound and MRI, the diagnosis was mesenchymal hepatic sarcoma. The paediatric patient had surgery to remove the entire liver segment containing the tumour. Micropathological examination showed that the tumour was a MHL. The serum AFP level fell rapidly to near normal following the surgery. The MHL benign liver tumour with an atypical presentation caused a very high AFP level. This was a rare clinical case, and it was difficult to diagnose.

## Introduction

Mesenchymal hamartoma of the liver (MHL) is a rare disease. This benign tumour usually occurs in children, from birth to 2 years.^1,2^

The oldest MHL cases were reported in 1959 and 1966. Additional cases have been reported until 2012. In total, less than 200 cases have been reported in the English literature.^3^

MHL is a benign developmental malformation composed of a mixture of loose mesenchymal tissue, bile ducts, connective tissue and hepatocytes with cysts formed either from degenerative areas of mesenchymal or from dilated bile ducts and lymphatics. It is not a true tumour.^4^

The tumour is usually in the form of a large cyst, with macrolobulated margin, multiple septae or a dense tumour containing of small cysts.^5^

In most MHL cases, α fetoprotein (AFP) levels are within the normal limits. Only in a few cases with increased AFP, it is easily confused with hepatoblastoma. Very few reports of AFP level above 6000 ng ml^−1^ have been reported in the medical literature.^3^

We report a special clinical case of a MHL of a 3-month-old paediatric patient with very high AFP level.

## Case report

A 3-month-old male paediatric patient was with a normal birth to full term. He was breastfed by his mother and weighted at 7.2 kg. His mother was healthy and there was no known liver disease in his family.

One month before his hospitalization, the patient went to a newborn check-up at the paediatric hospital. The result of the check-up uncovered a 50 mm damaged liver and then a very high AFP level. He was suspected of having hepatoblastoma. Liver function tests were normal and serology tests for hepatitis B and C were negative. Wako test was carried out at Medic Medical Centre, AFP level was very high, at 6388.4 ng ml^−1^. AFP-L3 and PIVKA (DCP) were within the normal limits, (AFP-L3 <0.5% and DCP was at 15 mAU/mL) (Table 1).

**Table 1.** HCC Risk Test (WAKO)

HCC Risk (WAKO)	18 March 2020	25 Apri 2020	9 June 2020	8 July 2020
AFP (ng ml^−1^)	6388.4	2176.4	74	44.4
AFP - L3 (%)	<0.5	<0.5	<0.5	<0.5
PIVKA II (DCP) (mAU ml^−1^)	15	14	24	22

**Figure 1.**
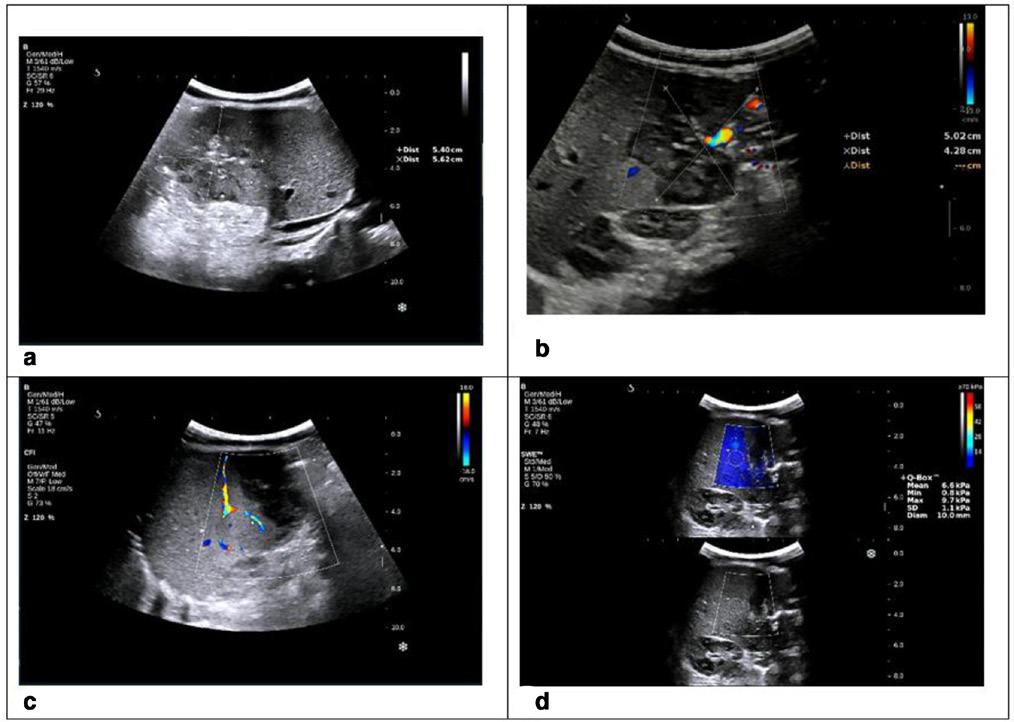
Abdominal ultrasound revealed a right hepatic lesion with mixed echo, multi curvilinear, (a, b) Mode ultrasound section showed right hepatic lesion, segment VI with many echogenic septae inside, b&c: Doppler ultrasound showed solid content with vessels, d: Real-time 2D shearwave elastography (SSI) showed solid content with low stiffness around 6.6 kPa.

At that time, an abdominal ultrasound revealed a right hepatic lesion, located in the segment VI, measuring 56 × 54 mm. The mixed echo cystoid tumour was unclearly delineated, multi curvilinear, with a solid content, hyper-echogenicity, echogenic vasculature and liquid with many echogenic septa inside. The solid content had low stiffness around 6.6 kPa in real-time 2D shearwave elastography. (Figure 1).

**Figure 2.**
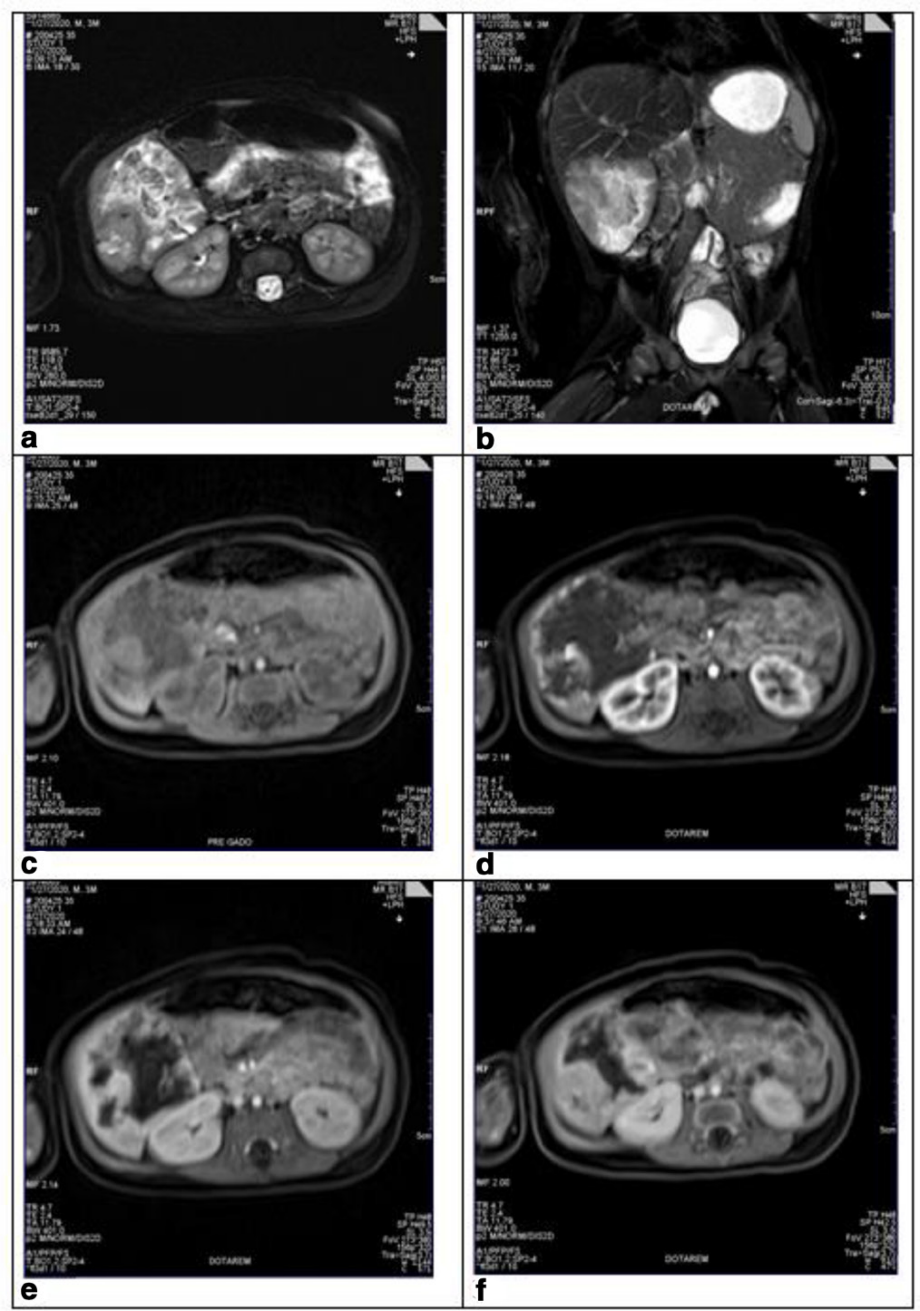
Contrast-enhanced MRI detected a right hepatic lesion in the segment VI. (a and b) The lesion had heterogeneous high signal intensity in axial and coronal T2W. c: The lesion has heterogeneous low signal intensity in T1W and T1 pre gadolinium. d: Heterogeneous gadolinium contrast opacification, peripheral enhancement of the arterial phase. (e and f) Contrast opacification progressive centripetally in the venous phase (T1 portal venous phase) and late phase (T1 delay phase) and without filling the whole lesion.

Contrast-enhanced MRI detected a right hepatic lesion in the segment VI, measuring 56 × 53 mm. The lesion had heterogeneous high signal intensity to the liver parenchyma in T2W, heterogeneous low signal intensity in T1W, heterogeneous gadolinium contrast opacification, peripheral enhancement of the arterial phase, progressive centripetally in the venous phase and late phase without filling the whole lesion. Delayed phase was taken through dynamic T1WI vibe pulse with contrast agent, which was recorded after 5–7 min from beginning of artery phase. (Figure 2).

The blood test revealed decreased AFP from 6388.4 to 2176.4 ng ml^−1^. AFP L3 and DCP remained within normal limits (Table 1).

Ten days later, the patient underwent surgery at the paediatric hospital, segment VI containing the tumour was removed and a biopsy of the lymph node of the hilum liver was performed. Histopathology; tumour was 4 × 5 cm, firm, and had hyper-generation of blood vessels.

**Figure 3.**
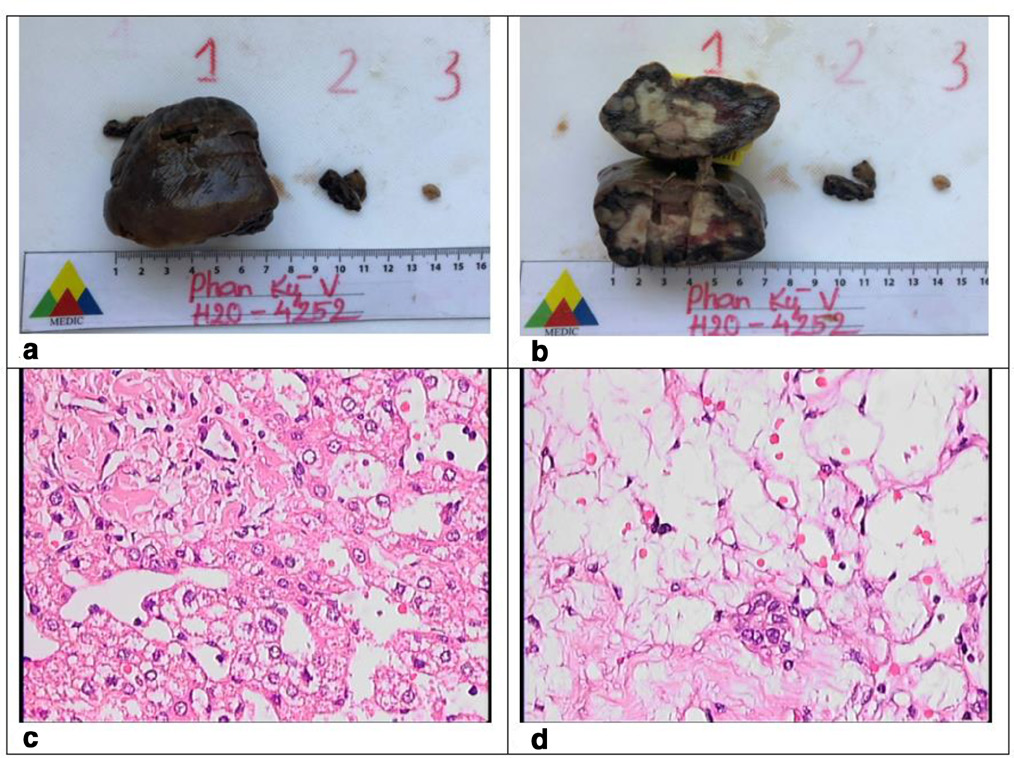
(a) and (b): Macropathology. (c) and (d): Micropathology.

Micropathology showed the specimen composed of cords of normal hepatocytes with loose cellular parenchyma, congestive blood vessels and hyalinized fibrous tissue. The microscopical diagnosis of the lymph node was a normal lymph node. Confirming a diagnosis of MHL (Figure 3).

**Figure 4.**
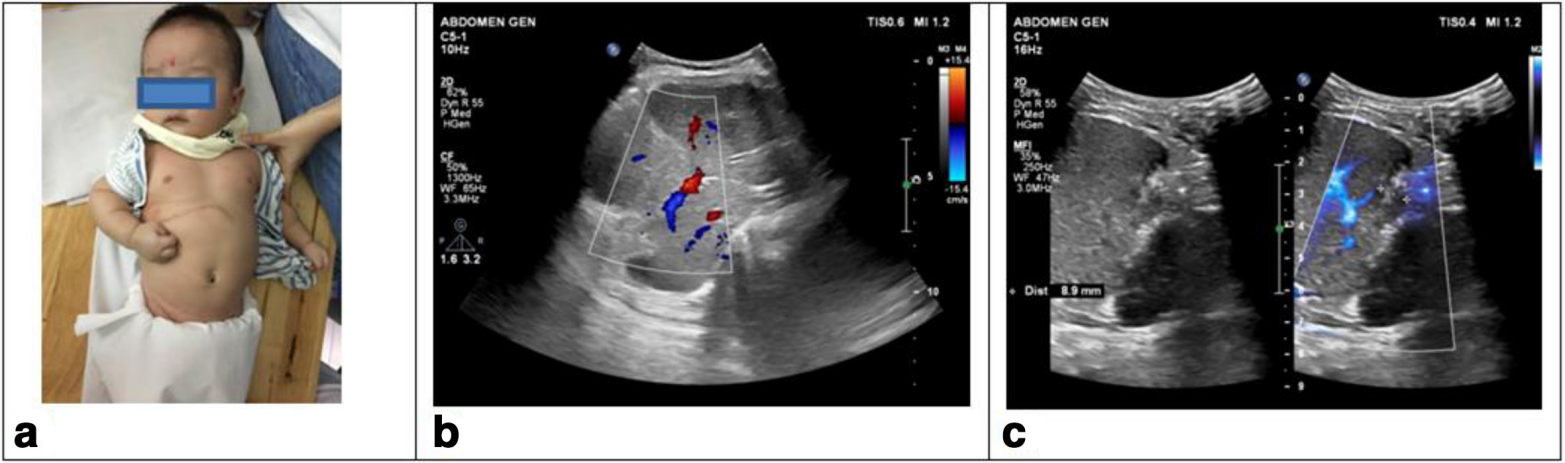
Pictures of the paediatric patient and abdominal ultrasound after the surgery.

One month after the surgery, the child was healthy, ate normally, gained weight steadily from 7.2 to 7.8 kg. Abdominal ultrasound showed the surgical scar healed well, fluid did not accumulate and tumour recurrence was not found. The portal vein and hepatic vein showed normal diameter and flow; there was no thrombosis (Figure 4). Blood test revealed a significant decrease of AFP level, at 74 ng ml^−1^. AFP-L3 <0.5% and DCP at 24 mAU/mL (Table 1).

Two months after the surgery, abdominal ultrasound showed the surgical scar well healed, and no recurrence of the tumour was found. The AFP continued to decline to close to the normal limit, at 44.4 ng ml^−1^ (Table 1).

## Discussion

MHL is a rare disease. This benign tumour usually occurs in children, between birth and 2 years of age,^1^ and ranks third among benign tumours in children after hemangioma and focal nodular hyperplasia (FNH).^5^ MHL is rare in adults. Commonly, males are more affected in children while females are more affected in adults.^1,6^

The oldest MHL cases were reported in 1959 and 1966. Additional cases have been reported until 2012. In total, less than 200 cases have been reported in the English literature.^3^

These tumours often have many cysts, ranging in size from a few millimetres to more than 10 cm, and are heterogeneous filled with a thick fluid. However, they may also be solid or of mixed forms. Ultrasound, CT and MRI showed images of a large cystic liver tumour, with a scalloped border, multiple septa or a solid mass of small cysts. The solid tissue composition of the tumour was revealed after the contrast agent injection.^5^

Radiographically, MHL is easily misdiagnosed as mesenchymal hepatic sarcoma. However, mesenchymal hepatic sarcoma is very rare, with clinical manifestations of abdominal pain, weakness, loss of appetite and weight loss. Mesenchymal hepatic sarcoma often shows signs of vascular invasion and biliary obstruction.^7,8^

AFP is a plasma glycoprotein that has a high level in fetal blood, is relatively high in newborns and gradually decreases to normal. M. E. C. Blohm et. al. analysed 524 samples who were aged from infants to 2 years at Düsseldorf University Hospital (Germany). At birth, the mean serum AFP level was 41687 ng ml^−1^ in 256 normal infants. AFP levels decreased by 50% after the first 5 days, then continued to decrease to 16–1995 ng ml^−1^ (average 178 ng ml^−1^) by 2 months of age and to 6–1045 ng ml^−1^ (average 80 ng ml^−1^) at 3 months of age. From 6 months to 2 years, AFP ranged from 0.8 to 87 ng ml^−1^ (average 8 ng ml^−1^).^9^

In most MHL cases, the AFP is within the normal range. Only a few cases demonstrated increased AFP, so it was easily confused with hepatoblastoma. Very few reports of an increase in AFP above 6000 ng ml^−1^ have been reported in the medical literature.^3,10^

A.Habador et. al reported a case of mesenchymal hepatic hamartoma, which was diagnosed as hepatoblastoma before surgery. The author believes that mesenchymal hamartoma and hepatoblastoma are common liver tumours in children. They have similar geometrical and pathological features. If the serum AFP is measured in a patient, an accurate diagnosis can be made before surgery, since AFP is largely elevated in hepatoblastoma. When in doubt, serum AFP is a good guide for differentiating hepatoblastoma from mesenchymal hamartoma.^11,12^

In this case study, the AFP was very high. Before surgery, AFP level was 6388.4 ng ml^−1^. After 1 month, there was a decrease, but it was still at a high level at 2176.4 ng ml^−1^. AFP decreased rapidly in 1 month after the surgery removed the whole segment containing the tumour, at 74 ng ml^−1^. Two months after the surgery, AFP continued to decrease to near the normal limit at 44.4 ng ml^−1^. It showed that the increase in AFP in this patient was related to the benign MHL tumour. AFP L3 and PIVKA II (DCP) were in normal range before and after the surgery.

Increased AFP and AFP-L3 are indicators for more accurate diagnose of malignant liver tumours for the newborn.^13^

## Conclusion

Mesenchymal hepatic hamartoma is a rare benign tumour that sometimes increases AFP level in the blood, causing a misdiagnosis of hepatoblastoma.

MHL usually presents as a large cystic, multilobulated, multi-septae or solid tumour with small cysts inside. The solid tissues of the tumour were revealed after enhancement by a contrast agent injection.

On imaging diagnose, MHL was misdiagnosed as mesenchymal hepatic sarcoma. To diagnose more accurately, we need to look for signs of vascular and biliary tract invasion in order to differentiate between MHL and mesenchymal hepatic sarcoma.

## Learning points

1. It is very difficult to diagnose liver tumours in children. The diagnosis should rely not only on imaging results but also many other factors.
2. In the event of a mismatch between the imaging results and the tumour markers, a liver biopsy should be performed.
3. Before performing surgery, the patient should be monitored to ensure the malignant diagnosis is correct. If the tumour markers decrease, a diagnosis of benign tumour should prevail.

## References

[1] Stocker JT, Ishak KG. Mesenchymal hamartoma of the liver: report of 30 cases and review of the literature. Pediatr Pathol 1983; 1: 245–67. doi: 10.3109/15513818309040663 6687279

[2] Atas E, Demirkaya M, et al. Mesenchymal hamartoma of the liver mimicking hydatid cyst. Pediatr Therapeut 2012; 2. doi: 10.4172/2161-0665.1000120

[3] Gaxa L, Hlatshwayo B. Mesenchymal hepatic hamartoma associated with elevated alpha fetoprotein mimicking a hepatoblastoma: a rare case and **a** literature review. Case Reports International 2017; 6: 9–12. doi: 10.5348/crint-2017-34-CR-3

[4] Anil G, Fortier M, Low Y. Cystic hepatic mesenchymal hamartoma: the role of radiology in diagnosis and perioperative management. Br J Radiol 2011; 84: e91–4. doi: 10.1259/bjr/41579091 21511744 PMC3473652

[5] Chung EM, Cube R, Lewis RB, Conran RM, et al. From the archives of the AFIP: pediatric liver masses: radiologic-pathologic correlation part 1. benign tumors. Radiographics 2009; 30: 801–26. 10.1148/rg.303095173 20462995

[6] Klaassen Z, Paragi PR, Chamberlain RS. Adult mesenchymal hamartoma of the liver: case report and literature review. Case Rep Gastroenterol 2010; 4: 84–92. doi: 10.1159/000260183 21103233 PMC2988903

[7] Luis Martí-Bonmatí MD, Ferrer D, Menor F, Galant J. Hepatic mesenchymal sarcoma: MRI findings. Abdominal Imaging 1993; 18: pages176–9. 10.1007/BF00198058 8439759

[8] Chen J, Du Y-J, Song J-T, E L-N, Liu B-R. Primary malignant liver mesenchymal tumor: a case report. World J Gastroenterol 2010; 16: 5263–6. doi: 10.3748/wjg.v16.i41.5263 21049562 PMC2975099

[9] Blohm ME, Vesterling-Hörner D, Calaminus G, Göbel U. Alpha _1_-fetoprotein (AFP) reference values in infants up to 2 years of age. Pediatr Hematol Oncol 1998; 15: 135–42. doi: 10.3109/08880019809167228 9592840

[10] Arrunategui AM, Caicedo LA, Thomas LS, Botero V, García O, Carrascal E, et al. Giant mesenchymal hamartoma in pediatric patients: a new indication for liver transplantation. J Pediatr Surg Case Rep 2017; 21: 1–3. doi: 10.1016/j.epsc.2017.03.012

[11] Bahador A, Geramizadeh B, Rezazadehkermani M, Moslemi S. Mesenchymal hamartoma mimicking hepatoblastoma. Int J Organ Transplant Med 2014; 5: 78–80. 25013683 PMC4089336

[12] Verma D, Agarwal S, Puri V, Singh D, Bundela T, et al. Mesenchymal hamartoma mimicking hepatoblastoma: a cytological pitfall. Journal of Cytology 2015; 32: 197–200. doi: 10.4103/0970-9371.168903 26729984 PMC4687214

[13] Kinoshita Y, Tajiri T, Souzaki R, Tatsuta K, Higashi M, Izaki T, Kinostuta YK, et al. Diagnostic value of lectin reactive alpha-fetoprotein for neoinfantile hepatic tumors and malignant germ cell tumors: preliminary study. J Pediatr Hematol Oncol 2008; 30: 447–50. doi: 10.1097/MPH.0b013e31816916ad 18525461

